# Basilar Artery Fenestration Aneurysm Treated With the Woven EndoBridge (WEB) Device: A Case Report and Review of the Literature

**DOI:** 10.7759/cureus.80517

**Published:** 2025-03-13

**Authors:** Albatool M Mansouri, Wesam O Mandoura, Jehad K Alharbi, Renad A Alsharif, Naif Alharbi

**Affiliations:** 1 Department of Neurosurgery, Al-Noor Specialist Hospital, Makkah, SAU; 2 College of Medicine, Umm Al-Qura University, Makkah, SAU; 3 College of Medicine, University of Jordan, Amman, JOR; 4 Department of Neurology, King Fahad General Hospital, Jeddah, SAU

**Keywords:** aneurysm, basilar artery, fenestration, subarachnoid hemorrhage, web, woven endobridge

## Abstract

Fenestration of the basilar artery (BA) is a rare congenital malformation resulting from anomalies in embryonic vascular development. Basilar artery fenestration aneurysms (BAFAs), especially in the proximal region, present unique challenges in neurosurgery. Their location in the critical and eloquent brainstem area makes open surgical clipping high-risk. Endovascular management has emerged as a less invasive alternative for aneurysms in such locations. The Woven EndoBridge (WEB) device (Terumo Neuro, Aliso Viejo, CA, United States) is a self-expanding, intrasaccular flow disruptor designed to treat wide-necked aneurysms without the need for additional devices, leading to improved outcomes and fewer complications from further interventions. This case describes a 52-year-old female patient with hypertension who presented to the emergency room following a two-minute seizure attack, preceded by a severe headache that began an hour earlier. Investigations revealed fenestration of the proximal BA trunk alongside a ruptured aneurysm at the fenestration set and diffuse subarachnoid hemorrhage (SAH). Successful embolization was performed using the WEB device, followed by flow diversion. BAFAs are rare vascular anomalies, and due to their critical location, careful assessment and treatment selection are crucial to avoid further complications. The WEB device offers a successful and viable treatment option, providing favorable outcomes with fewer complications.

## Introduction

The basilar artery (BA) is formed by the joining of two vertebral arteries at the medullo-pontine junction. It gives rise to three cerebellar arteries and two posterior cerebral arteries before terminating in the posterior cerebral circulation [1,2]. Fenestration of the BA results from the incomplete fusion of the longitudinal neuronal arteries, creating an artery with two lumens and possibly two adventitia [3-5]. With an approximate prevalence of 1%, the BA is the second most common site of cerebral arterial fenestrations, following the anterior communicating artery (AComm) [4-6]. These fenestrations typically occur in the proximal part of the BA trunk, just above the vertebrobasilar junction, and have been associated with aneurysm development at the fenestrated segments [3,4,7,8].

Proximal BA aneurysms present a unique challenge in neurointerventional surgery. Their location in the critical and eloquent brainstem area makes open surgical clipping high-risk. Over the past two decades, endovascular embolization has emerged as a minimally invasive alternative for the treatment of these complex lesions. From coil embolization, whether balloon or stent assisted, to flow diverters and flow disrupters, numerous techniques can be used in these cases.

Here, we present the case of a 52-year-old female patient with a fenestration of the proximal BA trunk and a ruptured aneurysm at the fenestrated segment, accompanied by diffuse subarachnoid hemorrhage (SAH). The aneurysm was successfully embolized using the Woven EndoBridge (WEB; Terumo Neuro, Aliso Viejo, CA, United States) intrassacular flow disruption device, followed by the deployment of a flow diverter. We also review the current literature on endovascular embolization for basilar artery fenestrated aneurysms (BAFAs) using the WEB device, alongside published reports of similar cases.

## Case presentation

**Figure 1:**
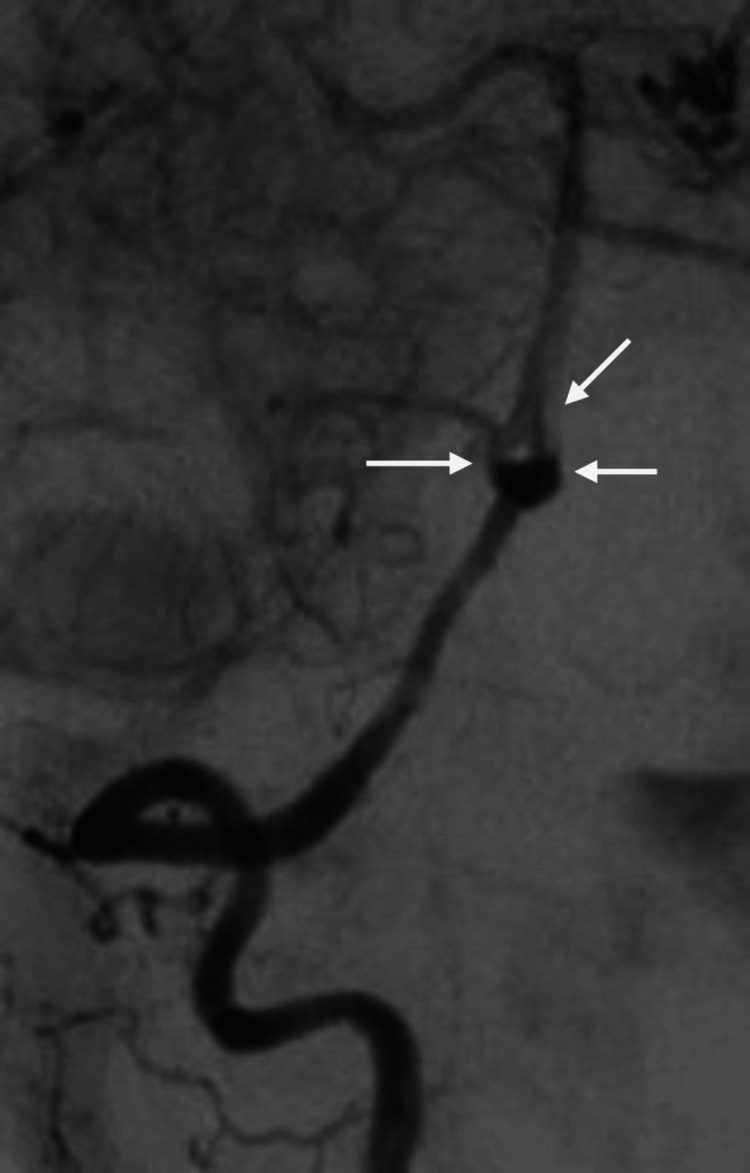
DSA pre-op showing fenestration of the basilar artery (white arrows) DSA: digital subtraction angiography.

**Figure 2:**
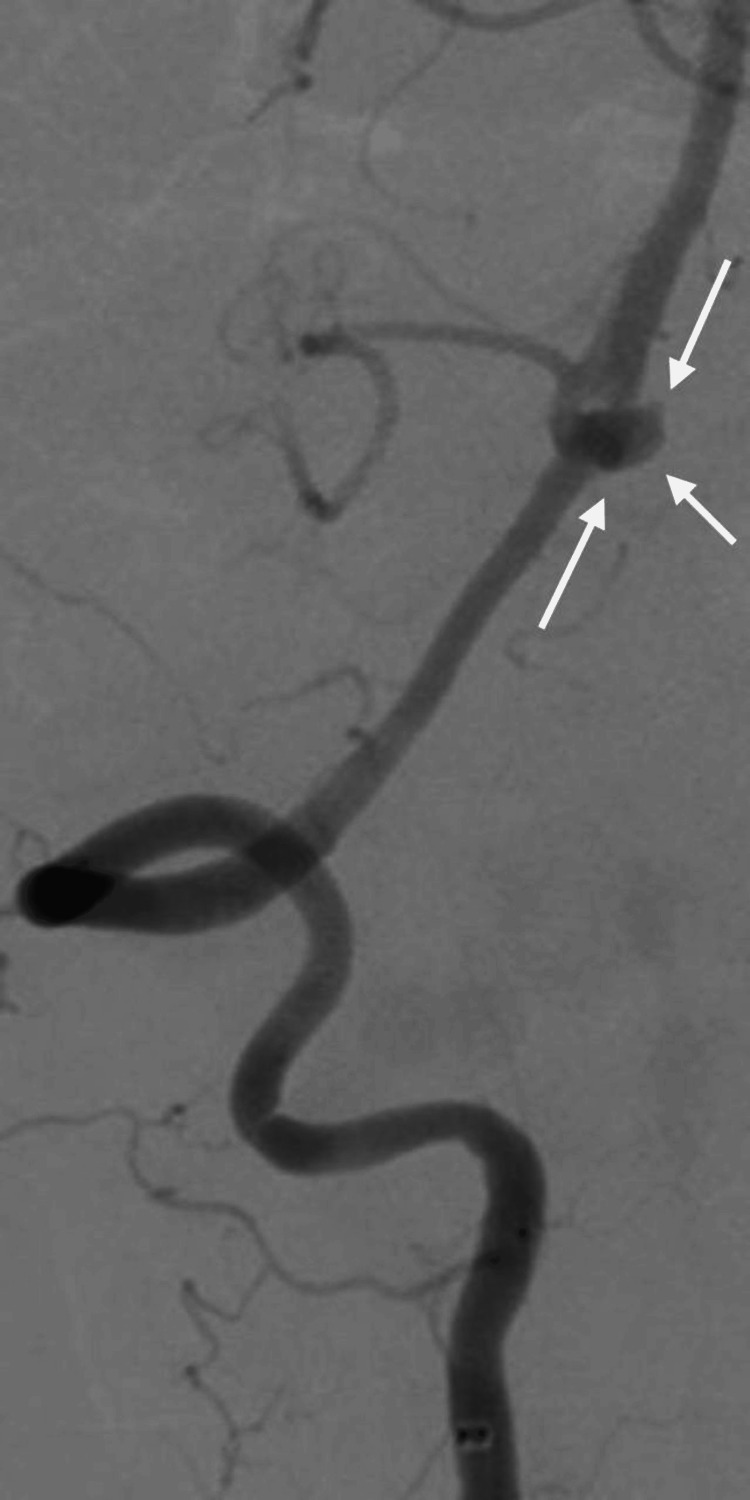
DSA pre-op showing aneurysmal sac out of the fenestration (white arrows) DSA: digital subtraction angiography.

**Figure 3:**
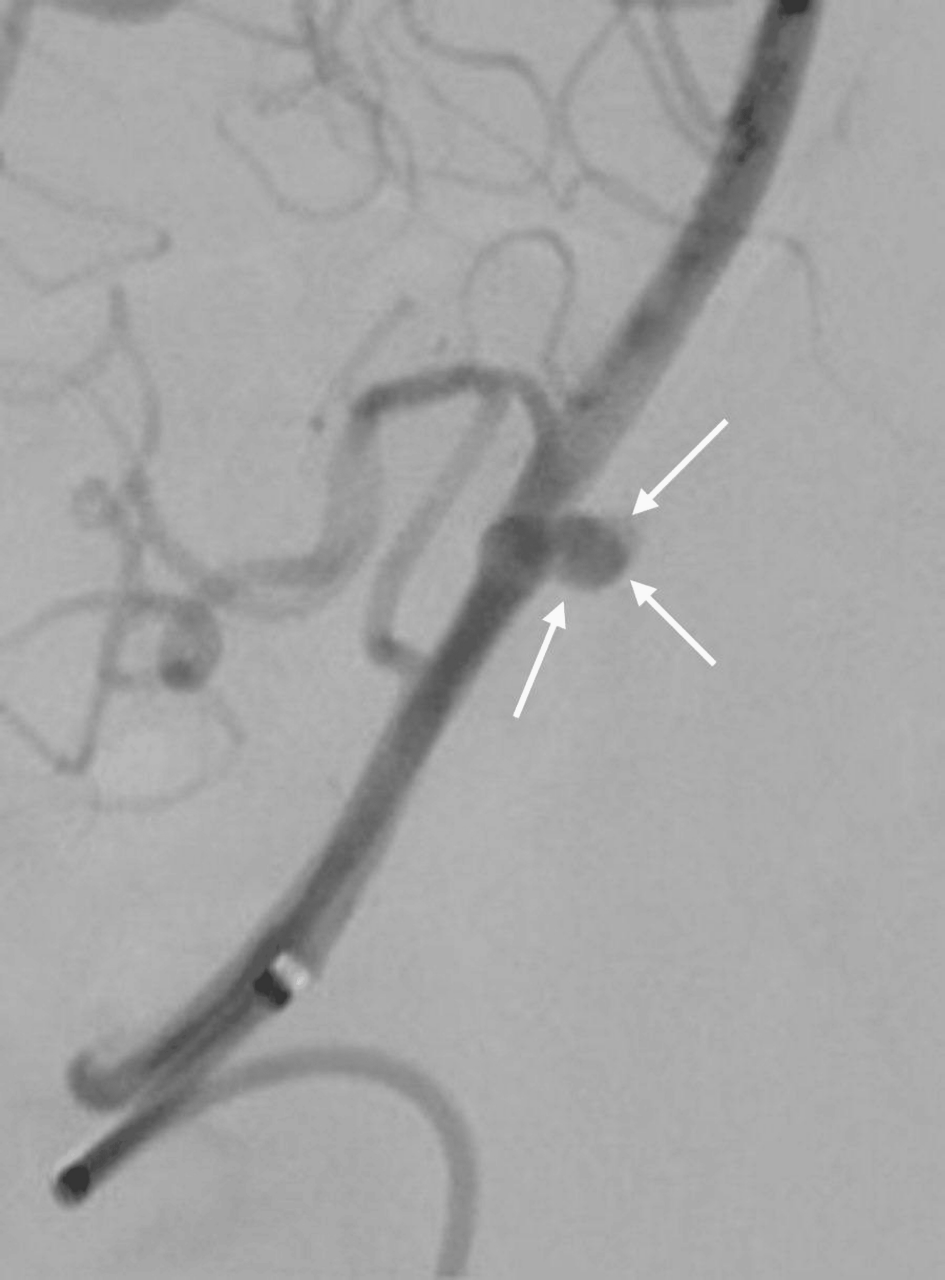
DSA pre-op showing an oblique view of the aneurysm (white arrows) DSA: digital subtraction angiography.

A 52-year-old female patient with hypertension presented to the emergency room following a 2-minute seizure attack, preceded by a severe headache that began an hour earlier. She also experienced palpitations and chest tightness. Upon presentation, the Glasgow Coma Scale (GCS) was 8/15, Hunt and Hess (H&H) IV, and World Federation of Neurological Surgeons (WFNS) IV. The pupils were 3 mm in size, equal, and reactive bilaterally. The patient exhibited more movement on her left side than on the right, with all brainstem reflexes intact. Computerized tomography (CT) and CT angiography (CTA) of the brain revealed fenestration of the BA trunk, alongside a ruptured basilar tip aneurysm associated with diffuse subarachnoid hemorrhage (SAH) and intraventricular hemorrhage, with a modified Fisher (mFISHER) grade II. In addition, the patient developed non-communicating hydrocephalus and underwent external ventricular drainage (EVD) insertion. The patient was transferred to our hospital for neurointerventional management. Digital subtraction angiography (DSA) confirmed a BA deformity (fenestration) (Figure 1) with an outpouching involving the anterior part of the proximal BA, likely representing a saccular aneurysm just above the vertebrobasilar junction (Figure 2 and Figure 3). The neck of the aneurysm measured 2 mm, and the sac measured 4 x 4 mm in the anteroposterior (AP) and transverse (TR) planes. 

**Figure 4:**
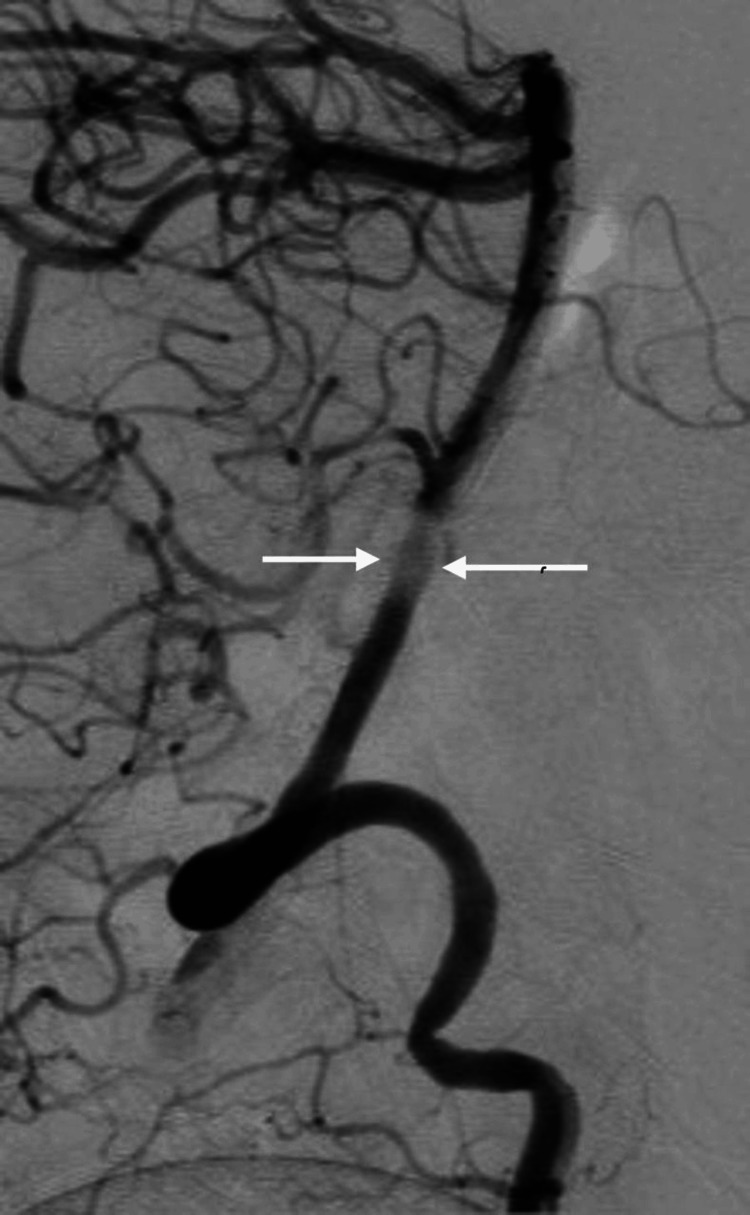
DSA after flow diversion installment (white arrows) DSA: digital subtraction angiography.

After a multidisciplinary meeting, it was decided to use the WEB device for an endovascular approach. Under sterile aseptic technique and general anesthesia, a 6-French sheath and catheter were inserted through the right femoral artery, with continuous flushing using heparinized saline. Under fluoroscopic guidance, with the primary feeding artery coming from the right vertebral artery, the main findings included BA fenestration with an intrasaccular aneurysm, as well as an incidental middle cerebral artery (MCA) trifurcation. The Traxcess system (Terumo Neuro, Aliso Viejo, CA, United States) was then used for catheterization and delivery of the WEB device, which measured 2 x 3.2 mm. After the device was deployed, a 10-minute angiography before detachment showed successful embolization of the aneurysm without complications. The arteriotomy was sutured using the ProGlide closure device (Abbott Laboratories, Chicago, IL, United States), followed by manual compression for 10 minutes. The patient was placed on aspirin 81 mg daily, in addition to the SAH protocol. Twenty-one days later, the patient underwent diagnostic DSA, which revealed significant recanalization of the aneurysm at the fenestration set. A second therapeutic DSA was performed the following day, and successful embolization of the proximal BAFA was achieved using flow diversion (Figure 4).

The patient was discharged on dual antiplatelet therapy (aspirin 81 mg and clopidogrel 75 mg daily) for three months, with no active issues. No seizures or neurological deficits were reported at the one-month follow-up. Aneurysm assessment with cerebral angiography is planned after two months.

## Discussion

**Figure 5:**
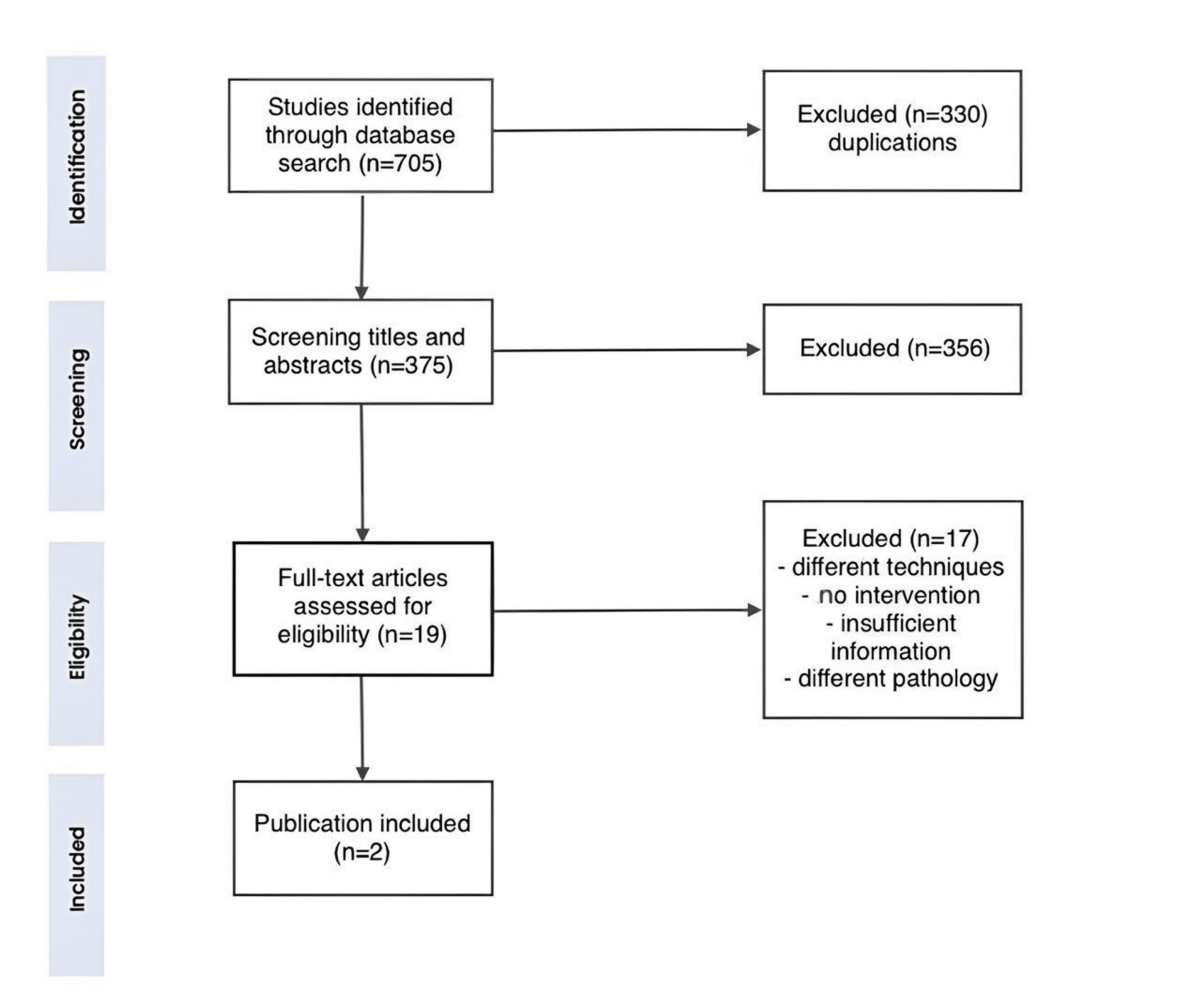
PRISMA flowchart PRISMA: Preferred Reporting Items for Systematic Reviews and Meta-Analyses.

Following the Preferred Reporting Items for Systematic Reviews and Meta-Analyses (PRISMA) guidelines, we systematically reviewed the literature related to BAFA on PubMed, Medline, and Web of Science (WoS) using the following search terms: ("fenestration" OR "fenestrated" OR "fenestrations") AND ("basilar" OR "BA") AND ("aneurysm" OR "aneurysms"). This search included studies published up to August 2024. We also screened the references of the included studies for possible relevant articles. We included case reports, case series, case-control studies, and observational studies published in English. Eligible articles met the following criteria: (1) patients with BAFAs, whether ruptured or unruptured, (2) endovascular treatment of BAFAs using the WEB device, (3) sufficient reporting of aneurysm morphology, (4) angiographic results pre- and postoperatively, and (5) reporting of complications peri- or postoperative complications. We excluded abstracts, conference papers, animal studies, review articles, and studies using treatment methods other than WEB, and articles with insufficient reporting of aneurysm morphology or treatment modality (Figure 5).

Two authors independently screened titles and abstracts, followed by a full-text screening of the included studies, with a third reviewer resolving any discrepancies.

BA fenestration is an anatomical variant in which the artery forms two lumens, each lined with muscular and endothelial layers, and may involve the adventitia. This phenomenon occurs due to the failure of fusion of the primitive longitudinal neuronal arteries during embryonic development [3,6,7]. BA fenestration is the second most common site of cerebral arterial fenestrations after AComm. However, it is easier to detect on angiography than AComm due to its larger size [4-6]. While BA fenestrations can occur at any part of the trunk, those in the proximal segment, just above the vertebrobasilar junction, are the most common [3,4,7]. This involvement of the lower half of the vessel is believed to be due to the cranio-caudal fusion of the longitudinal arteries during development [7].

Aneurysms associated with BA fenestration have been widely reported [3-6,8-10], especially in the proximal segment [3,8,11]. Aneurysms arising from the proximal BA, defined as those distal to the origin of the anterior inferior cerebellar artery, are particularly challenging due to their proximity to eloquent brainstem structures and the BA bifurcation. The walls of the fenestrated limbs have normal architecture laterally, but medially, there is an elastin discontinuity at the proximal part, along with subendothelial thinning [3,8,12]. These structural changes are also observed in cerebral arterial bifurcations. Furthermore, high blood flow and excessive pressure on the fenestration wall lead to hemodynamic instability, significantly increasing the risk of aneurysm formation [3,4,8].

Surgically approaching aneurysms in such a location while minimizing complications presents a challenge. The approach requires cranial access to the lower part of the brainstem, an area containing the lower cranial nerves and numerous small arterial perforators. This complex anatomy makes successful microsurgical clipping difficult [4,8,11]. Several endovascular techniques have been described for treating proximal BA aneurysms, including coil embolization, balloon-assisted coil embolization, stent-assisted coil embolization, flow diversion, and parent vessel occlusion [4,8]. The choice of technique depends on the aneurysm size, morphology, neck width, and the presence of branches incorporated into the aneurysm dome [6,8,13]. To our knowledge, this is the third reported case, alongside Drijkoningen et al. [10] and de Almeida Silva et al. [4], of treating an aneurysm at the tip of a fenestrated BA using the WEB device, meeting the inclusion criteria outlined earlier. Table 1 displays data from two previous cases, in addition to the current study, including demographics, clinical presentation, aneurysm morphology, and patient procedural and clinical outcomes. The study by Styczen et al. [14] also reports the treatment of BAFA with endovascular techniques including WEB. However, it did not provide sufficient information to meet the criteria for our study.

**Table 1:** Table of included articles and their data ASA: acetylsalicylic acid, BA: basilar artery, CT: computed tomography, DM: diabetes mellitus, DSA: digital subtraction angiography, HTN: hypertension, MRI: magnetic resonance imaging, UFH: unfractionated heparin, WEB: Woven EndoBridge.

Author	de Almeida Silva et al. [4]	Drijkoningen et al. [10]	Current study
Year	2021	2021	2024
Demographics			
Sex	Female	Female	Female
Age in years	75	66	52
DM	No	No	No
HTN	Yes	No	Yes
Smoking	Yes	No	No
Clinical presentation			
Presenting symptoms	Headache	Headache and nausea	Headache and seizure
Initial imaging	CT	CT	CT
Confirmatory imaging	DSA	DSA	DSA
Aneurysm morphology			
Site	Lower one-third of the BA	Vertebrobasilar junction	Proximal tip of the BA
Morphology	Sac and neck	Blood blister-like aneurysm	Sac and neck
Sac size	5.5 x 5.5 mm	2 mm	4.4 mm
Neck size	5.2 mm	-	2 mm
Rupture	No	Yes	Yes
Procedure			
Preop medications	ASA 200 mg/day	No	No
Peri-op medication	Heparinization with 5,000 IU of UFH	Heparinization with 3,000 IU of UFH	Heparinization with 3,000 IU of UFH
Postop medication	No	ASA 81 mg	ASA 81 mg
WEB size	6 x 3 mm	4.4 x 2.6 mm	2 x 3.2 mm
Complications			
Mortality	No	No	No
Recurrence	No	No	No
Retreatment	No	No	Yes
New neurological deficits	No	No	No
Follow-up			
Duration (mean)	6 m	3 m	1 m
New neurological deficits	No	No	No
Angiography	6 m	-	-
MRI	-	3 m	-
Occlusion	Yes	Yes	-

The WEB device is an intrasaccular flow disruptor featuring a self-expanding braided mesh made of platinum-cored nitinol wires, delivered via a micro-catheter. Upon deployment, the mesh covers the aneurysm neck, achieving metallic coverage ranging from 55% to 100%. This coverage disrupts intra-aneurysmal blood flow, promoting intrasaccular thrombosis and promoting neo-endothelialization [4,15]. Specifically designed to treat wide-necked aneurysms without the need for additional supporting devices [15], the WEB device has been successfully used in a wide range of aneurysms, regardless of neck size, morphology, rupture status, or location [15-17]. A case reported by Drijkoningen et al. [10] highlighted a unique blood blister-like aneurysm in a BA fenestration, where the sac was smaller than the neck in both lobes; despite this, the WEB device was still effectively used. In a meta-analysis by Asnafi et al. [17], the initial occlusion rate for wide-neck bifurcation aneurysms was higher in patients treated with the WEB device (60%) compared to those treated with stent-assisted coiling (54%). Furthermore, the need for pre- and/or perioperative antiplatelet therapy is reduced when using a WEB device, especially for patients with ruptured aneurysms, as demonstrated in the current case and the case reported by Drijkoningen et al. [10].

In the current case, although the neck of the aneurysm was narrow (2 mm), significant recanalization occurred after the initial occlusion was achieved. This was subsequently treated successfully with flow diversion. This was subsequently treated successfully with flow diversion. Such recanalization is considered one of the less significant complications of using the WEB device, often requiring retreatment with another WEB device, flow diversion, or coiling [14,17]. Factors associated with recanalization include large aneurysm size, wide neck, and incomplete initial occlusion. Asnafi et al. [17] reported retreatment rates of 4% for ruptured aneurysms and 7% for unruptured aneurysms treated with the WEB device, similar to the rates published by van Rooij et al. [15,16]. Although device deployment carries the risks of treatment failure, thromboembolic complications, and retreatment, these risks are still lower compared to other endovascular techniques like coiling [15-18]. On the other hand, angiographic and clinical outcomes after WEB application are promising. Numerous retrospective case series have reported high rates of immediate aneurysm occlusion with endovascular WEB treatment and even higher rates of mid- to long-term occlusion of 70%-96%. Furthermore, good midterm neurological outcomes were achieved in the vast majority of the cases [15-17]. Despite these positive outcomes and advances in endovascular technology, several technical challenges remain. Navigating guidewires and catheters through the tortuous vertebrobasilar system can be difficult, with risks of perforator occlusion or brainstem infarct. Therefore, careful patient selection, accurate device measurements, precise deployment, antiplatelet therapy, and close follow-up are crucial to minimize complications.

## Conclusions

The field of endovascular neurointervention is continuously evolving, with new devices and techniques being developed. The use of intrasaccular flow disruptors has the potential to improve angiographic outcomes and reduce complications. However, further prospective studies with larger sample sizes and long-term follow-up are needed to compare the safety and efficacy of different endovascular techniques and to optimize patient outcomes. A multidisciplinary approach involving neurointerventionists, neurosurgeons, and neurologists is essential for optimal patient selection and management.
